# Effects of a novel peptide Ac-SDKP in radiation-induced coronary endothelial damage and resting myocardial blood flow

**DOI:** 10.1186/s40959-018-0034-1

**Published:** 2018-12-18

**Authors:** Umesh C. Sharma, Swati D. Sonkawade, Andrew Baird, Min Chen, Shirley Xu, Sandra Sexton, Anurag K. Singh, Adrienne Groman, Steven G. Turowski, Joseph A. Spernyak, Supriya D. Mahajan, Saraswati Pokharel

**Affiliations:** 1 Department of Medicine, Division of Cardiology, Jacob’s School of Medicine and Biomedical Sciences, Buffalo, NY USA; 2 Laboratory Animal Shared Resource Facility, Roswell Park Cancer Center, Buffalo, NY USA; 3 Department of Radiation Oncology, Roswell Park Comprehensive Cancer Center, Buffalo, NY USA; 4 Department of Biostatistics and Bioinformatics, Roswell Park Comprehensive Cancer Center, Buffalo, NY USA; 5 Translational Imaging Shared Resources, Roswell Park Comprehensive Cancer Center, Buffalo, NY USA; 6 Department of Medicine, Jacob’s School of Medicine and Biomedical Sciences, Buffalo, NY USA; 7 Department of Pathology, Division of Thoracic Pathology and Oncology, Roswell Park Comprehensive Cancer Center, Buffalo, NY 14203 USA

**Keywords:** Ac-SDKP, Cardiac MRI, Endothelial cells, Fibrosis, Radiation

## Abstract

**Background:**

Cancer survivors treated with thoracic ionizing radiation are at higher risk of premature death due to myocardial ischemia. No therapy is currently available to prevent or mitigate these effects. We tested the hypothesis that an endogenous tetrapeptide N-acetyl-Ser-Asp-Lys-Pro (Ac-SDKP) counteracts radiation-induced coronary vascular fibrosis and endothelial cell loss and preserves myocardial blood flow.

**Methods:**

We examined a rat model with external-beam-radiation exposure to the cardiac silhouette. We treated a subgroup of irradiated rats with subcutaneous Ac-SDKP for 18-weeks. We performed cardiac MRI with Gadolinium contrast to examine resting myocardial blood flow content. Upon sacrifice, we examined coronary endothelial-cell-density, fibrosis, apoptosis and endothelial tight-junction proteins (TJP). In vitro, we examined Ac-SDKP uptake by the endothelial cells and tested its effects on radiation-induced reactive oxygen species (ROS) generation. In vivo*,* we injected labeled Ac-SDKP intravenously and examined its endothelial localization after 4-h.

**Results:**

We found that radiation exposure led to reduced resting myocardial blood flow content. There was concomitant endothelial cell loss and coronary fibrosis. Smaller vessels and capillaries showed more severe changes than larger vessels. Real-time PCR and confocal microscopy showed radiation-induced loss of TJ proteins including- claudin-1 and junctional adhesion molecule-2 (JAM-2). Ac-SDKP normalized myocardial blood flow content, inhibited endothelial cell loss, reduced coronary fibrosis and restored TJ-assembly. In vitro, Ac-SDKP localized to endothelial cells and inhibited radiation-induced endothelial ROS generation. In vivo*,* labeled Ac-SDKP was visualized into the endothelium 4-h after the intravenous injection.

**Conclusions:**

We concluded that Ac-SDKP has protective effects against radiation-induced reduction of myocardial blood flow. Such protective effects are likely mediated by neutralization of ROS-mediated injury, preservation of endothelial integrity and inhibition of fibrosis. This demonstrates a strong therapeutic potential of Ac-SDKP to counteract radiotherapy-induced coronary disease.

**Electronic supplementary material:**

The online version of this article (10.1186/s40959-018-0034-1) contains supplementary material, which is available to authorized users.

## Introduction

The success of cancer radiotherapy, developed over decades for the most aggressive forms of cancers, has led to the application of this treatment modality to more than 50% of the cancer patients. [1–3]. With millions of patients surviving cancer over the last decade, use of radiotherapy is a clinical reality, raising hope for patients with aggressive and life-threatening tumors [4]. However, premature coronary artery and cardiovascular diseases have created an unanticipated risk of late morbidity and mortality [5]. The only available clinical solution is “*dose reduction*” of radiation, which may risk local control of the cancer. Consequently, a large proportion of patients with thoracic and mediastinal cancers, including those of the lung, esophagus and stomach, thymomas and lymphomas receive considerable radiation doses [6–8]. Despite increased morbidity and mortality associated with radiation-induced cardiovascular disease, no specific therapeutic agents are available to-date to prevent or mitigate these conditions.

Prior studies have examined the detrimental effects of ionizing radiation in endothelial cell integrity [9, 10]. This is expected since endothelial cells are the most radiosensitive cells originating from mesenchyme [11, 12]. Ionizing radiation can induce oxidative damage to the endothelial cells both directly and via paracrine mechanisms. This can alter the endothelial cell physiology including blunted vasodilatation response, reduced capillary formation and loss of cell permeability [9, 13]. Endothelial cells express tight junctions (TJ) proteins, which are formed by different components. At TJ, cell attachment results from the protein members of claudin and occludin family, which are strongly associated with other intracellular proteins such as junctional adhesion molecule 2 (JAM-2) and zonula occludens-1 (ZO-1) [14, 15]. These junctional elements are essential to maintain the integrity of the vessel wall. Modifications of the expression of these proteins by ionizing radiation can have complex downstream effects on the vascular function.

Studies by our group and others have shown anti-inflammatory and anti-fibrotic effects of a novel peptide, N-acetyl-Ser-Asp-Lys-Pro (Ac-SDKP or Seraspenide) [16, 17]. In 2008, Lin and associates demonstrated that Ac-SDKP prevented vascular inflammation and reactive oxidative injury in a rat model of angiotensin II infusion [18]. Our initial studies in renal capillary (glomeruli) of hypertensive mice showed protective effects of Ac-SDKP against inflammation, fibrosis and albuminuria [19]. While Ac-SDKP-dependent reconstitution of TJ molecules could be speculated, no confirmatory data exist to date to explain the anti-proteinuric effects of Ac-SDKP. Expression of tight-junction molecules was initially examined by Hajem et al in human epidermal cells. Ac-SDKP was reported to improve the epidermal barrier by stimulating the expression of protein components of tight junctions including claudin-1, occludin and zonula occludens-1 (ZO-1), thus playing an important role in connecting neighboring cells [20]. Despite these important effects of Ac-SDKP in preserving the epithelial barrier, there are no studies that have examined the role of Ac-SDKP in endothelial cell integrity.

Ac-SDKP is a tetrapeptide generated by the hydrolysis of its precursor, thymosin β_4_ [21]. Ac-SDKP is thought to be synthesized in bone marrow and mononuclear cells, and is found in various organs including heart, kidneys and blood vessels [17, 19, 21]. We have previously reported that Ac-SDKP strongly inhibits inflammation and fibrosis in pre-clinical models of cardiac fibrosis [17]. In our recently published study, we demonstrated novel effects of Ac-SDKP for the inhibition of myocardial macrophage infiltration and galectin-3 expression [22]. Despite a wealth of information regarding anti-inflammatory and anti-fibrotic effects of Ac-SDKP, there are no data in the literature that determines whether this novel molecule can be used for the inhibition of radiation-induced endothelial damage and vascular fibrosis.

In this study, we hypothesized that radiation-induced coronary vascular damage and subsequent loss of myocardial blood flow is mediated by oxidative damage to the endothelial cells, and the disruption of endothelial TJ assembly. This leads to altered endothelial physiologic response including loss of cell continuity and reduced expression of major cell membrane barrier proteins. Such effects can lead to loss of structural integrity and altered immune responsiveness of the endothelial cells consequently leading to vascular damage and fibrosis. Here, we report that a novel endogenous tetrapeptide, Ac-SDKP promises strong therapeutic benefits by i) attenuating radiation-induced reactive oxidative stress (ROS), ii) inhibiting endothelial cell loss, iii) protecting endothelial tight junction assembly, and iv) inhibiting coronary arterial fibrotic remodeling, and thus preserving myocardial blood flow. These data are of important therapeutic implications since there are no available therapies for radiation-induced vascular damage. Ac-SDKP has been shown to be a safe endogenous peptide that has demonstrated no side effects, at least in the preclinical models.

## Materials and methods

### Experimental groups

The animal care and experimental protocols followed US National Institutes of Health guidelines and were approved by the Institutional Animal Care and Use Committees of the Roswell Park Comprehensive Cancer Center (RPCCC) at Buffalo, NY. The experimental design for radiation exposure including the safety data for 30 Gy of radiation and 3.2 mg/kg/day of peptide therapy has been recently reported by our group in a cardiomyopathy-focused study [22]. Briefly, age-matched Sprague Dawley (SD) rats were used. Isoflurane anesthetized rats (10–12 week old) received a single dose of 30 Gray (Gy) radiation or Sham therapy into the left hemithorax (which spans whole heart, left mediastinum) using an orthovoltage radiator (Philips, Best, the Netherlands). Rats received experimental radiation with 250 kV photons with a 1-cm cone. Surrounding tissue was covered with 2-mm thick lead plates to limit unintended radiation exposure. Rats were observed during radiation exposure and for at least 30 min into recovery.

Fourteen rats were treated with continuous subcutaneous infusion of Ac-SDKP (3.2 mg/kg/day) for 18 weeks using ALZET® Mini-Osmotic Pumps (infusion was started within 24 h of radiation exposure). Twelve additional age-matched radiation unexposed rats were used as biological controls. After the completion of 18-week treatment protocol, animals were sacrificed (with CO_2_ overexposure) and organs were harvested for additional histopathological and molecular analysis.

In addition to the rat study protocol, we used 4 mice (C57BL/6 background, age 16–18 weeks) for in vivo Ac-SDKP peptide imaging protocol.

### Resting myocardial blow flow

Following acquisition of three baseline SPGR images (Echo time = 2.2 ms, flip angle = 90°, matrix =128 × 128, FOV = 6.0 × 6.0 cm, 2 mm slice thickness, 4 averages), resting myocardial blood flow imaging was performed in mid-ventricular short-axis orientation after 0.3 mmol/kg of gadolinium (Gd-DTPA) injection via tail vein catheter. Images were acquired using the pulse sequence described previously [23, 24]*.* Myocardial signal intensity during the contrast distribution was divided by the signal intensity of posterior chest wall muscles and the average normalized ratio was reported as the resting myocardial blood flow as described previously by Friedrich and associates [25]. The optimal signal intensities were measured at the mid-left ventricular myocardium (average time for hyperemia: 2–3 min). Repetition time (TR) was controlled by cardiac rate, which was maintained at an R-R interval between 170 and 200 ms and semiquantitative data analysis was performed using an in-house developed image analysis algorithm written in MATLAB (Mathworks, Nattick, MA) by a data processor blinded to experimental groups. Small number of rats with skin reactivity against minipumps or those showing suboptimal chest wall signal intensities were non-selectively excluded from the study.

### Coronary vascular fibrosis

For the evaluation of coronary vessels, 5-μm-thick transmyocardial sections were obtained from formalin-fixed paraffin embedded heart tissue. Trichrome staining was used to evaluate the extent of vascular and peri-vascular fibrosis, which included medium to small coronary vessels in the myocardium. Fibrosis quantification was performed on multiple images obtained from at least 5 random tissue planes from each animal. The total vascular area and the area of positive staining for fibrosis were quantified using NIH imaging software Fiji (National Institute of Health, Bethesda, MD, USA).

### Measurement of endothelial cell density

Luminal endothelial cells were counted in medium to small coronary vessels in hematoxylin and eosin-stained endothelial monolayers according to the study protocol described previously [26]. The total number of endothelial cells were normalized by the mean cross-sectional area of the corresponding blood vessels.

### Normalization of the endothelial cell numbers and vascular fibrosis to coronary luminal size

The coronary luminal size was determined using the measurement tool on the Fiji Software [27]. Luminal area, measured in pixels^2^, was multiplied by the adjusted conversion factor of 8.26 pixels^2^/μm^2^ to interchange between units of pixels^2^ and μm^2^. Since larger vessels tended to have a larger amount of fibrosis compared to smaller vessels, the fibrotic areas of the coronary arteries were normalized by the luminal area. The endothelial cells (number of cells/μm^2^) were similarly normalized to the luminal area using the same conversion factor described above.

### CD31 immunohistochemical staining and terminal deoxynucleotidyl transferase dUTP-nick end labeling (TUNEL) staining for apoptosis detection

The CD31 immunohistochemical staining was performed at the Roswell Park Comprehensive Cancer Center (*Pathology Resource Core Laboratory*) according to the manufacturer’s protocols. Briefly, 5 μm sections obtained from formalin-fixed paraffin embedded rat hearts were deparaffinized, rehydrated and blocked for endogenous peroxidase using 3% H_2_O_2_ in dark. The sections were incubated with rat monoclonal antibody against murine CD31 (Abcam #Ab64543) in a 1:50 dilution followed by incubation with secondary antibody. Finally, the sections were exposed to 3–3-diaminobezedine (DAB) and counterstained with hematoxylin. The images were obtained from at least ten random microscopic fields. The number of positive cells were counted by two independent and blinded observers.

For apoptosis detection, TUNEL assay was performed using S7101 ApopTag® Plus Peroxidase in Situ Apoptosis Kit (MilliporeSigma, Burlington, MA) in 4 μm formalin-fixed paraffin sections as described previously by our group [22].

### RNA isolation and real-time quantitative PCR

For the quantitative analyses of mRNA expression of tight junction molecules, we performed quantitative real time PCR for *claudin-1, claudin-3, claudin-5*, *JAM-2, occludin* and *ZO-1* on the rat heart tissue. The mRNA was isolated using E.Z.N.A.® Total RNA Kit I (Omega Bio-tek). The cDNA was synthesized using Verso cDNA Synthesis Kit (Thermo Fisher) according to the manufacturer’s instructions. In each qPCR reaction the cDNA equivalent of 5 ng mRNA was used. The qPCR reactions contained SsoAdvanced™ Universal SYBR® Green Supermix (Bio-Rad), and the forward and reverse primers were each added to a final concentration of 1 μM. The amplification protocol consisted of three steps: i) 95 °C for 30 s, ii) 39 cycles of 5 s amplifications at 95 °C, 30 s at 59 °C and 5 s at 95 °C and, iii) 65–95 °C melt curve, by 5 °C increments. The sequences of the primers used for real-time PCR are shown in Table 1. The housekeeping gene, GAPDH, was used as the internal control. In the comparative or ΔΔCt method of qPCR data analysis, the Ct values obtained from mRNA samples examined in duplicate are directly normalized to a housekeeping gene and then compared. This method assumes that the PCR amplification efficiencies of the gene of interest and the housekeeping genes are close to 100%. The difference between the Ct values (ΔCt) of the gene of interest and the housekeeping gene is calculated for each experimental sample, followed by calculating the difference in the ΔCt values between the experimental and control samples (ΔΔCt). The fold-change in expression of the gene of interest between the two samples is then equal to 1/2^(-ΔΔCt) and also expressed as transcript accumulation index (TAI) [28, 29].

**Table 1:** Primers used in the quantitative real time-PCR

Gene	Forward Primer 5′to 3′	Reverse Primer 5′to 3′	Length mRNA (bp)
*Claudin-1*	GGCCAGGCTCTCTTTACTGG	AGAGGTTGTTTTCCGGGGAC	93
*Claudin-3*	AAGGTGTACGACTCGCTGCT	AGTCCCGGATAATGGTGTTG	247
*Claudin-5*	GGGCGTCCAGAGTTCAGTTT	TAACTTGCCTCGGAGTCTGC	213
*JAM-2*	GGCGACTTTAAAGACCGTGC	AGTTGCGGGCTTCACAGTAA	412
*Occludin*	GGATTGAGCCCGAGTGGAAA	GAGGTAGCACCACGTTGGAA	400
*ZO-1*	GGGGCCTACACTGATCAAGA	GGTCTCTGCTGGCTTGTTTC	370

*JAM-2* junctional adhesion molecule 2, *ZO-1* zonula occludens

### Immunofluorescence and confocal microscopy

Formalin-fixed and paraffin-embedded rat heart sections were used for immunofluorescence-based staining. Antibody incubation was done after dewaxing and antigen retrieval according to the manufacturer’s instructions. Rat heart tissue was incubated with a rat-specific anti-Jam-2 (Abcam, Cambridge, MA; Ab139645) and anti-Claudin-1 (Abcam, Cambridge, MA; Ab15098) antibodies overnight followed by exposure to Alexa 594 conjugated secondary antibody. In addition, FITC labeled-peptide injected mice heart sections were stained with anti-Von Willebrand factor (Abcam, Cambridge, MA; Ab11713). Slides were counterstained with DAPI and coverslips were applied using ProLong™ Gold Antifade mounting media (Invitrogen, P36931). Images were captured via epifluorescence microscope (ZEISS LSM 800 Laser scanning microscope).

### Measurement of endothelial cell reactive oxidative stress

We performed ROS-Glo™ H2O2 assay (Cat No. G8820, Promega) to measure radiation-induced ROS generation in endothelial cells. The assay is based on a luminescent signal generated by a chemical reaction between H_2_O_2_ and its substrate. Briefly, HUVECs were seeded (10 × 10^4^ /well) on gelatin-coated NUnclon 96 Flat white plate (Thermo Fisher Scientific). After 24-h incubation, the medium was replaced and cells were irradiated (9Gy). A subgroup of cells was treated with Ac-SDKP (100 nM) immediately after radiation. These cells were then incubated for 18 h before a mixture of H_2_O_2_ substrate and H_2_O_2_ dilution buffer was added. After 6 h of incubation with the dilution buffer, ROS-Glo detection solution was used as directed. The luminescence was measured using a FL_X_800 microplate fluorescence reader (BIO-TEK instruments).

### Ac-SDKP cytochemistry, confocal microscopy and FACS analysis

To examine the uptake of Ac-SDKP by Human Cardiac Microvascular Endothelial Cells (HMVEC-Cs; Cat No. CC-7030, Lonza), we first performed in vitro cell imaging studies using fluorescein isothiocyanate (FITC) conjugated with Ac-SDKP. The FITC-conjugated Ac-SDKP was synthesized using Fmoc solid phase peptide synthesis (Genescript, Piscataway, NJ) and FITC-conjugated scrambled peptide, Ac-KDPS was synthesized using similar Fmoc synthesis protocol (Lifetein, Somerset, NJ). For these studies, peptide uptake was examined in both irradiated and non-irradiated (control) HMVEC-C cells at four hours post peptide treatment. For confocal image analysis, HMVEC-C cells were cultured on sterile cover slips in a 12 well plate. One of the plates was exposed to 9 Gy of radiation using a specialized orthovoltage radiation chamber and a sub group of cells were incubated with FITC-labeled Ac-SDKP or scrambled peptides at 14 μM. Images were captured using a confocal microscope (ZEISS LSM 800 Laser scanning microscope) at 400X magnification. For determining the uptake using FACs, cells were detached, irradiated and incubated with FITC-labeled scrambled peptide or Ac-SDKP (0.9, 1.8, 3.5, 7 & 14 μM). Cell uptake percentage was examined with a BD Accuri C6-Plus flow cytometer and further analyzed by FCS express 6-De Novo Software.

#### In vivo FITC-Ac-SDKP uptake protocol

The in vivo uptake study was performed in four C57BL/6 mice (Cyagen, Santa Clara, CA). Before the infusion of fluorescent peptide, mice received 30 Gy radiation exposure to the left hemithorax or underwent Sham therapy. After 24 h, 200 μM of FITC-labeled Ac-SDKP or FITC-labeled scrambled peptide was injected via the tail-vein in 200 μl of phosphate-buffered saline (PBS) solution using 28-gauge/0.5-in. insulin syringes (Becton Dickinson, Franklin Lakes, NJ). After 4 h, mice were sacrificed (with CO_2_ overexposure) and heart tissues were harvested. Isolated hearts were perfused with PBS, flash-frozen and cryo-sectioned (16 μm thickness) for further florescence image analysis.

#### Statistical analyses

Quantitative endpoints were summarized by group using the mean and standard error of the mean (SEM). When appropriate, the endpoints were modeled as a function of treatment group (control, radiation alone, and radiation + Ac-SDKP) using one-way ANOVA models; with between-group comparisons made using Tukey-Kramer adjusted post-hoc tests. The association between cellular uptake and Ac-SDKP peptide concentration was evaluated using the Spearman correlation coefficient. Unpaired t-test was done for group-wise comparison of myocardial blood flow. All model/test assumptions were verified graphically using quantile-quantile and residual plots, with transformations applied as appropriate. All tests were two-sided at a nominal significance level of 0.05; that is, *p*-values < 0.05 were considered significant.

## Results

### Normalization of resting myocardial blood flow content by Ac-SDKP therapy

**Fig. 1:**
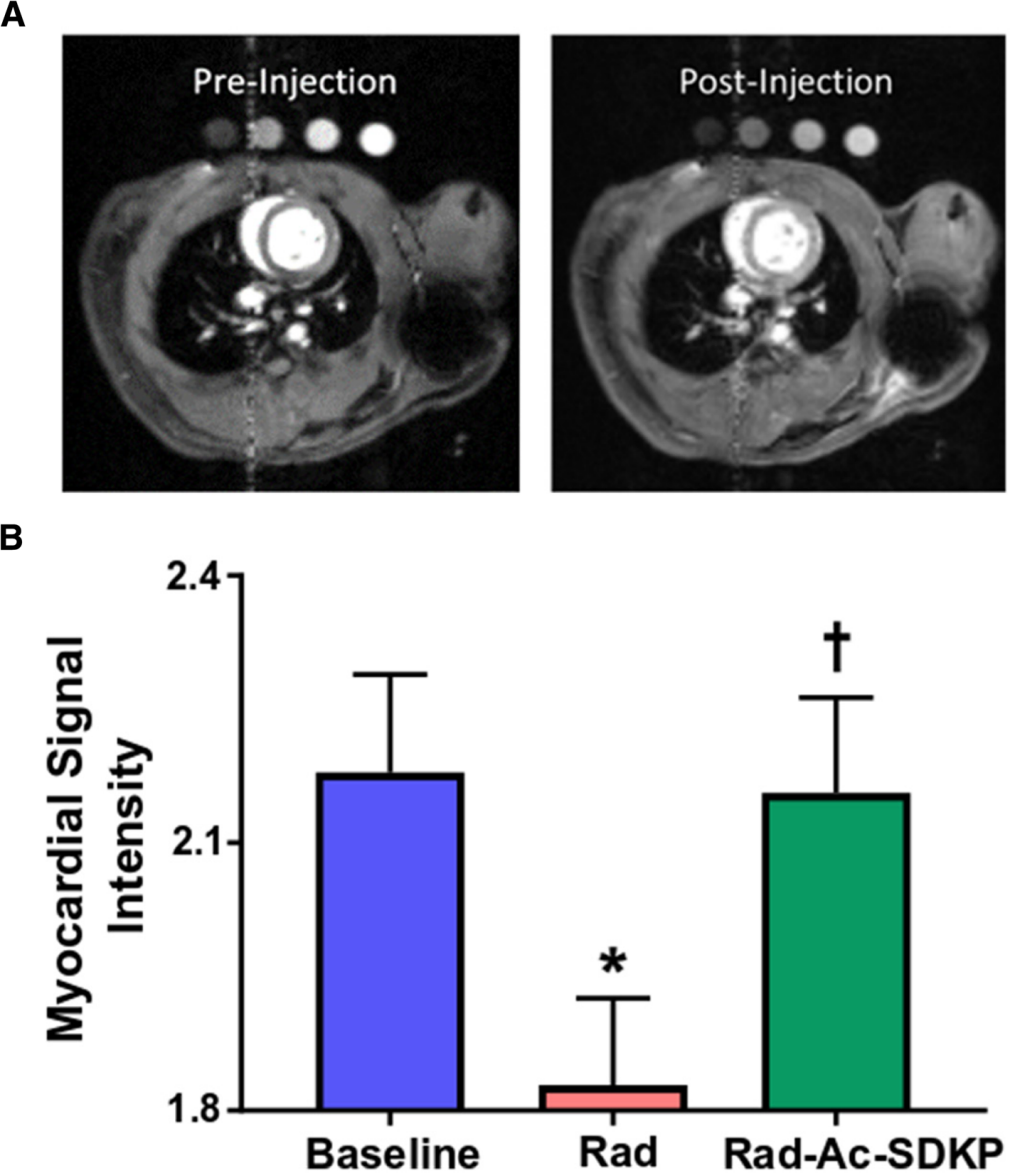
Quantification of myocardial blood flow content by gated cardiac MRI after Gadolinium contrast injection: **a** represents the increase of mid-myocardial signal intensities immediately (2–3 min) after intravenous gadolinium injection. **b** represents the early enhancement signal of the hyperemic myocardium, which was compared between three experimental groups. Signal intensity was quantified within specific regions of interest at the mid-myocardial segments. Reduction of the signal intensity was visualized in rats exposed to cardiac irradiation. Ac-SDKP normalized the signal intensities to baseline levels. *N* = 11–15 each group, *, *p* = 0.02 rad vs. baseline, †, *p* = 0.03 rad vs. rad + Ac-SDKP. Rad, radiation

The signal intensities immediately after the intravenous contrast injection were compared at the mid-myocardial levels using papillary muscles as the anatomical landmarks. Compared to non-radiated baseline controls, thoracic radiation exposure led to reduction of mid-myocardial blood flow content as demonstrated by reduced signal intensities after Gadolinium infusion (mid-myocardial signal intensity: baseline, 2.18 ± 0.10; radiation, 1.82 ± 0.09, *p* = 0.02, *N* = 14–15 per group) (Fig. 1 a & b). The signal intensity at the Y-intercept was comparable to those of normal controls in Ac-SDKP treated rats, which is consistent with preserved myocardial blood flow content after chronic Ac-SDKP therapy (mid-myocardial signal intensity: Ac-SDKP, 2.15 ± 0.10, *p* = 0.03 vs. radiation group, *N* = 11, Ac-SDKP therapy group).

### Inhibition of radiation-induced coronary vascular endothelial cell loss

**Fig. 2:**
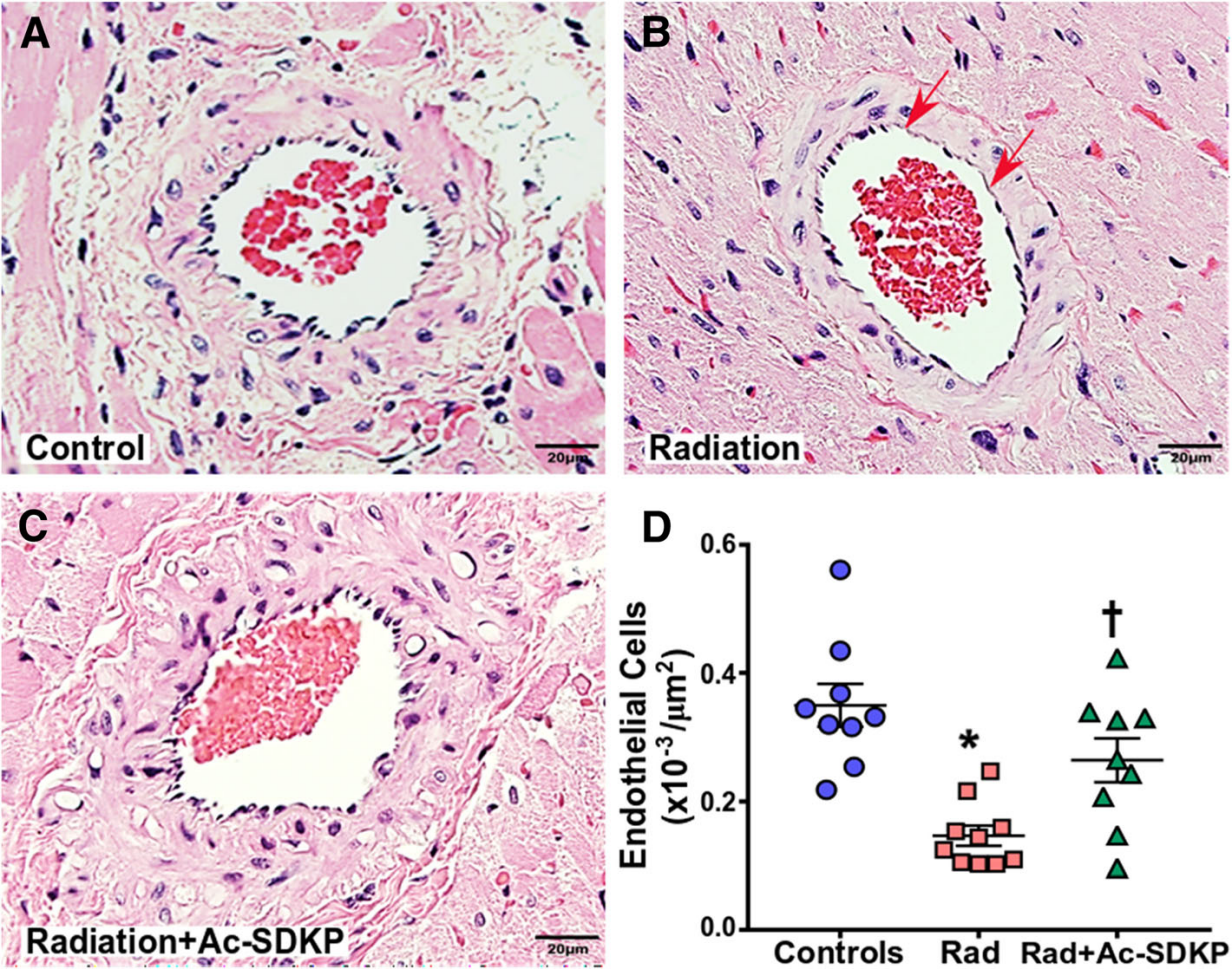
Effects of Ac-SDKP on endothelial cell denudation induced by radiation exposure: **a** to **c** show the representative images of coronary blood vessels identified in myocardial sections following hematoxylin and eosin staining. **a**: Non-radiated controls; **b**: Radiation exposed rats (arrows point to foci of endothelial cell dropout); and **c**: Radiation + Ac-SDKP treated rats. **d**: Quantification of coronary vascular endothelial cells in control and radiation exposed rats with and without Ac-SDKP therapy. *N* = 9–10 each group, *, *p* < 0.001 rad vs. control, †, *p* = 0.005 rad vs. rad + Ac-SDKP. Rad, radiation. Scale bar: 20 μm, magnification × 400

Histological analysis of coronary vessels from the control rats showed an intact endothelial cell lining (Fig. 2 a). In contrast, rats with radiation exposure showed intermittent loss of endothelial cells (Fig. 2 b). In particular, there was a significant drop of endothelial cells per cross sectional area in medium and small vessels of radiation-exposed rats. Rats treated with Ac-SDKP demonstrated preserved endothelial cell continuity in the coronary vessels (Fig. 2 c). These data implicate that Ac-SDKP protects against radiation-induced endothelial damage and/or enhances their recovery (endothelial cells × 10^− 3^/μm^2^: control, 0.35 ± 0.03; radiation alone, 0.15 ± 0.01; radiation + Ac-SDKP, 0.26 ± 0.03, *p* < 0.001 for control vs. radiation and 0.005 for radiation vs. radiation + Ac-SDKP, *N* = 9–10 each group) (Fig. 2 d). In addition, TUNEL staining on the myocardial specimens showed no evidence of endothelial cell apoptotic DNA fragmentation on either of the experimental groups (Additional file 1: Figure S1).

### Preservation of coronary microvascular density

**Fig. 3:**
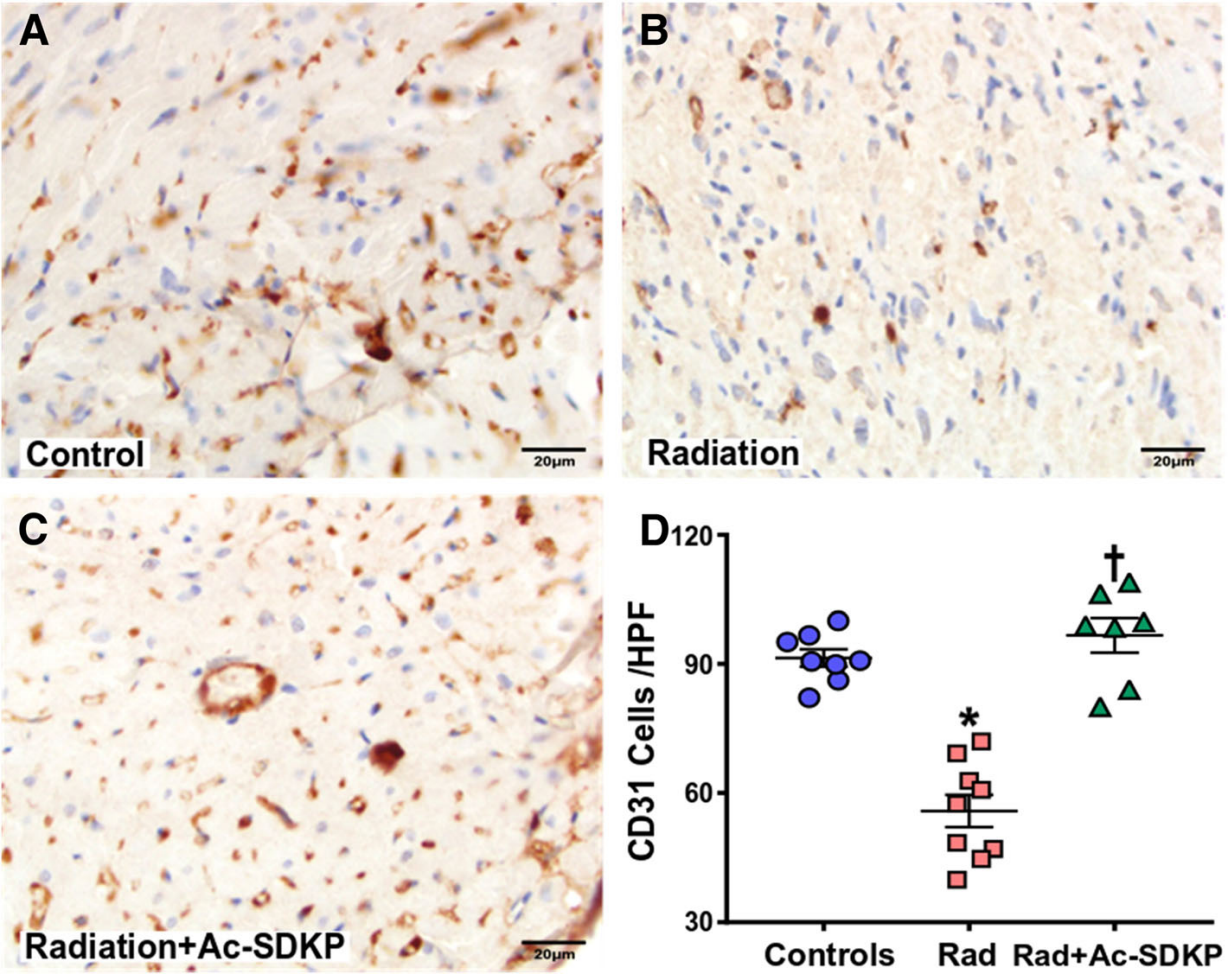
Assessment of myocardial capillary density: Panel **a** to **c** demonstrate the representative images of CD31-stained myocardial tissue sections. **a**: Non-radiated control; **b**: Radiation; **c**: Radiation + Ac-SDKP treatment. Endothelial cells are stained with brown staining. **d**: Quantification of CD31 positive cells in control and radiated rats with and without Ac-SDKP therapy. *N* = 7–10 each group, *, *p* < 0.001 rad vs. control, †, *p* < 0.001 rad vs. rad + Ac-SDKP. Rad, radiation. Scale bar: 20 μm, magnification × 400

We evaluated myocardial microvascular density by immunohistochemical staining with CD31 antibody, which reacts with endothelial cells (Fig. 3 a-c). Since capillaries constitute the majority of the endothelial cell volume, positive staining mostly represents microvascular endothelial cell density. Compared to controls, radiation exposure led to significant reduction of CD31 positive cells (CD31-positive cells/HPF: control, 91.4 ± 2.0 radiation, 55.8 ± 3.8, *p* < 0.001, *N* = 8–9 each group). Treatment with Ac-SDKP normalized the number of CD31 positive cells in irradiated rats (CD31-positive cells/HPF: radiation + Ac-SDKP, 96.7 ± 3.8, *p* < 0.001vs. radiation, *N* = 7) as shown in Fig. 3 d.

### Inhibition of the fibrotic remodeling of the coronary vessels

**Fig. 4:**
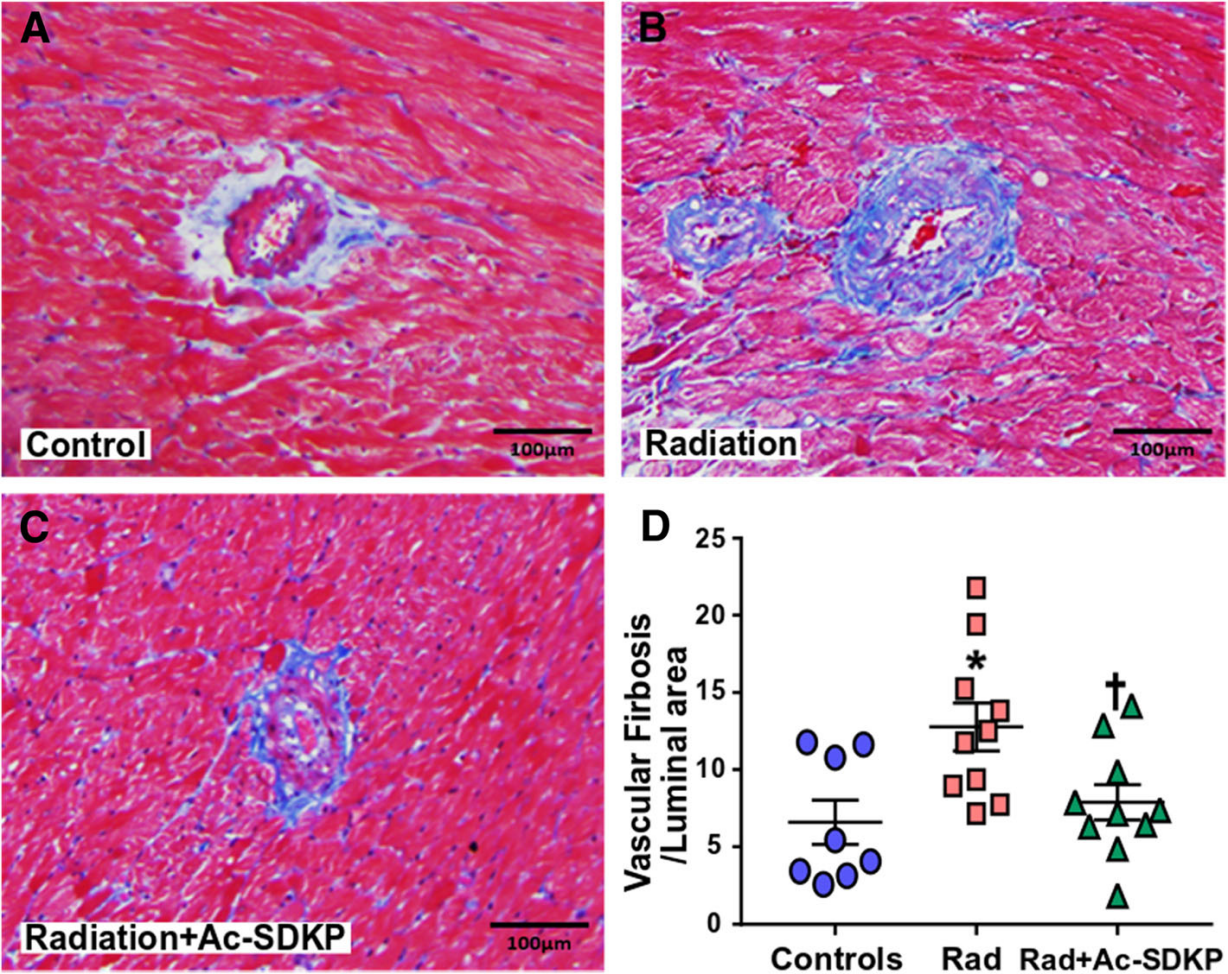
Effect of Ac-SDKP on coronary vascular fibrosis induced by radiation exposure: **a** to **c** show the representative images of Masson’s trichrome staining of rat coronary vessels in myocardial sections. **a**: Non-radiated baseline control; **b**: Radiation; and **c**: Radiation + Ac-SDKP treatment. Blue staining represents collagen. **d**: Quantification of perivascular collagen in control and radiation exposed rats with and without Ac-SDKP therapy. *N* = 8–10 each group, *, *p* = 0.01 rad vs. control, †, *p* = 0.04 rad vs. rad + Ac-SDKP. Rad, radiation. Scale bar: 100 μm, magnification × 100

Since radiation-induced injury to the coronary blood vessels can induce fibrosis, we performed quantitative evaluation of perivascular collagen content in Masson-trichrome-stained myocardial tissue sections (Fig. 4 a-c) 18 weeks after IR exposure. Compared to controls, rats subjected to irradiation showed a dense perivascular accumulation of collagen in medium to small coronary vessels (% fibrosis/luminal area: control, 6.6 ± 1.4; radiation, 12.8 ± 1.6, *p* = 0.01, N = 8–10 each group). These peri-vascular fibrotic changes were significantly lower in irradiated rats that were also treated with Ac-SDKP (% fibrosis/luminal area: radiation + Ac-SDKP, 7.9 ± 1.1, *p* = 0.04 vs. radiation, *N* = 10). (Fig. 4, d).

### Effects of radiation and Ac-SDKP therapy on cardiac endothelial tight-junction gene and protein expression

**Fig. 5:**
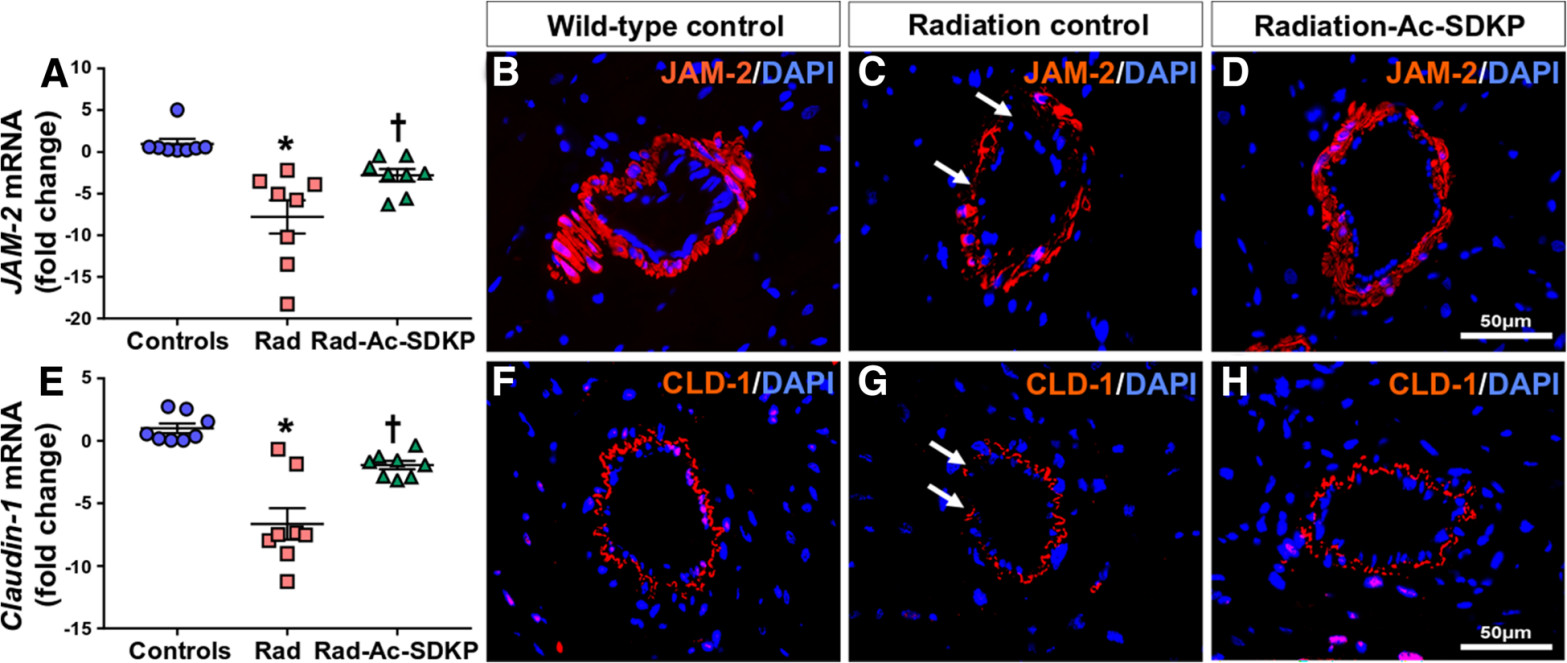
Effects of radiation and Ac-SDKP therapy on the mRNA and protein expression of tight junction molecules: Post-radiation rat heart tissue mRNA was analyzed by qPCR for measuring expression of common tight junction genes *JAM-2 and claudin-1.* Radiation exposure significantly reduced gene expression of *JAM-2* (**a**) and *Claudin-1* (**e**) in rat cardiac tissues (*, *p* ≤ 0.003 compared to controls), which was restored by Ac-SDKP treatment (†, *p* ≤ 0.03 compared to rad). *N* = 8–10 each group. **b-d**: Representative immunofluorescence images showing expression of intercellular cell adhesion proteins JAM-2 **(b-d**) and claudin-1 (**f-h**) into the rat coronary vessels. The immunofluorescence shows a well-defined TJP expression including, JAM-2 and claudin-1 (red stain). DAPI stained nuclei are shown in blue. **c** & **g** represent tissue sections from radiation exposed rats. Radiation exposed group showed patchy loss of TJP expression (*arrows*). Ac-SDKP reconstituted JAM-2 and claudin-1 expressions (**d** & **h**). Rad, radiation. Scale bar: 50 μm, magnification × 400

To evaluate the mRNA expression of transmembrane and adaptor tight-junction molecules, we performed quantitative real-time PCR for mRNA expression of *claudin-1*, *claudin-3, claudin-5, JAM-2, occludin* and *ZO-1* in cardiac tissue homogenates. Based on the relative threshold cycle values, we noted significant suppression of *JAM-2* (8 ± 2.0 fold lower than control, *p* = 0.003), *claudin-1* (7 ± 1.2 fold lower than control, *p* = 0.0001), *claudin-3* (3 ± 0.8 fold lower than control, *p* = 0.001), and *ZO-1* (5 ± 2.2 fold lower than control, *p* = 0.02) expression in radiation exposed rats. Ac-SDKP treatment significantly reversed the radiation-induced suppression of *JAM-2* (*p* = 0.03 vs. radiation) and *claudin-1* (p = 0.001 vs. radiation) mRNA expression (Fig. 5 a & e). Additionally, A-SDKP treatment showed a trend to restore radiation-induced loss of *clauidn-3* (*p* = 0.03 vs. radiation) and *ZO-1* (*p* = 0.07 *vs.* radiation) expression. Expression levels of *occludin* and *claudin-5* were not significantly affected by radiation or Ac-SDKP treatment (Additional file 2: Figure S2). In line with our RT-PCR data, compared to baseline controls (Fig. 5 b & f), confocal microscopy of the coronary vessels showed radiation-induced loss of JAM-2 and claudin-1 (Fig. 5 c & g), which was partially restored in Ac-SDKP treated groups (Fig. 5 d & h).

### Ac-SDKP inhibits radiation-induced intracellular reactive oxygen species generation in endothelial cells

**Fig. 6:**
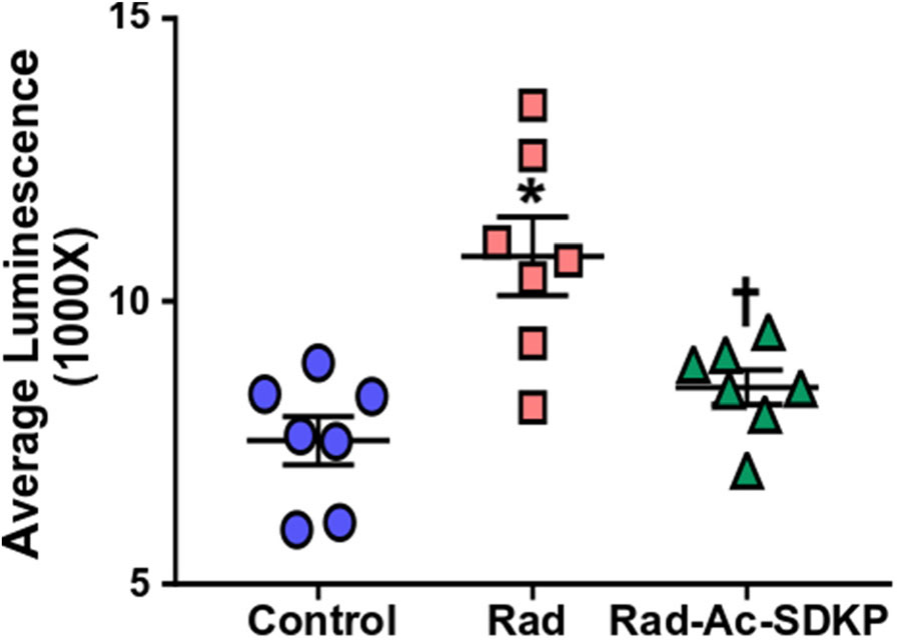
Effects of Ac-SDKP in radiation-induced intracellular ROS generation in endothelial cells: Luciferase light signal as a measure of reactive oxygen species (ROS) activity examined by ROS-Glo™ H_2_O_2_ Assay. Average luminescence signal from the ROS-Glo™ Assay was compared with baseline control and treated cells. When compared to baseline control, radiation exposure significantly increased ROS generation in endothelial cells, which was inhibited by Ac**-**SDKP treatment group. *N* = 7 each group, *, *p* < 0.001 rad vs. control, †, *p* = 0.01 rad vs. rad + Ac-SDKP. Rad, radiation

Radiation exposure to endothelial cells is known to generate ROS in the affected organs. We examined the ROS generating effects of radiation and the protective effects of Ac-SDKP in cultured HUVECs. Compared to control, radiation exposure led to significantly increased ROS generation in endothelial cells. Treatment of cells with Ac-SDKP at 100 nM inhibited the ROS generation (average luminescence × 1000: radiation, 10.8 ± 0.7; radiation + Ac-SDKP, 8.2 ± 0.2, *p* = 0.01, *N* = 7 each group) (Fig. 6).

### Ac-SDKP uptake by cultured HMVEC-Cs

**Fig. 7:**
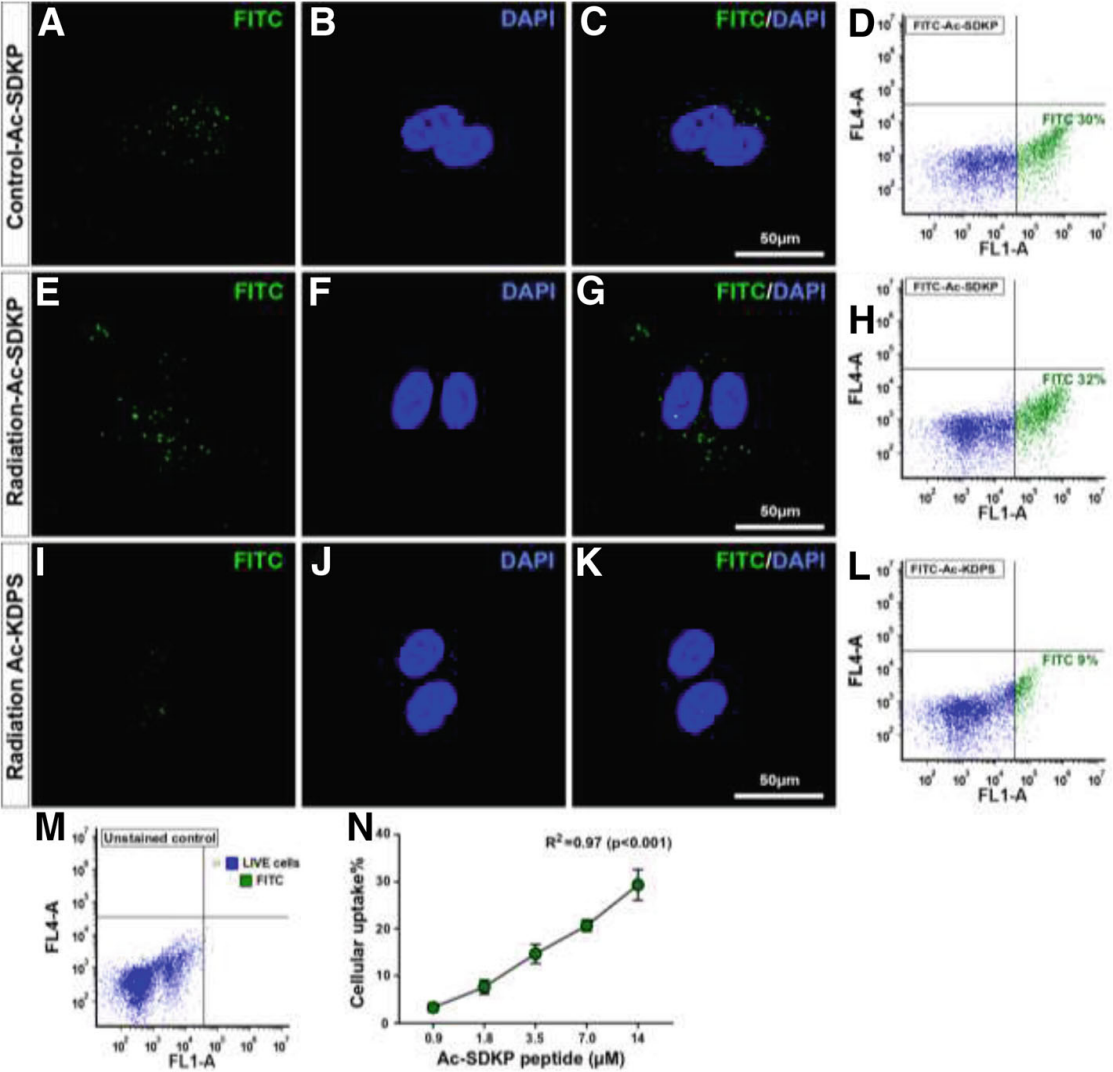
Uptake and localization of FITC-Ac-SDKP by HMVEC-Cs: Human cardiac microvascular endothelial cells (HMVEC-Cs) were treated with FITC-labeled Ac-SDKP and scrambled peptide immediately prior to radiation, and the cells were incubated for 4 h. **a**: Representative confocal image showing FITC-labeled peptide (green) localized in to the non-irradiated (control) endothelial cells; **b**: DAPI-stained nuclei (blue), and **c**: Merged FITC and DAPI images. **e-g** shows localization of FITC-Ac-SDKP in irradiated HMVEC-Cs. **i-k** shows weak, non-specific localization of scrambled peptide, FITC-Ac-KDPS, in irradiated HMVEC-Cs. **d**, **h** and **l** compares FACS results for FITC-Ac-SDKP in non-irradiated cells (30% uptake), FITC-Ac-SDKP in irradiated cells (32% uptake), and FITC-Ac-KDPS (scrambled peptide) in irradiated cells (9% uptake), respectively. The gating was set with FL1-H (FITC) positive cell population in green and negative cells in blue. **m** and **n**: Graphs showing representative irradiated unstained population used as gating reference (**m**) and dose-dependency of FITC-Ac-SDKP uptake by endothelial cells (**n**). *N* = 3 in each treatment. Scale bar:50 μm, magnification × 400

To study cell-type specific uptake and localization of Ac-SDKP, we synthesized fluorophore-conjugated Ac-SDKP peptide and a control scrambled peptide, allowing visualization of peptide uptake. Confocal microscopy generated three dimensional images of the fluorescent peptide, illustrating a robust uptake of FITC-Ac-SDKP into the HMVEC-Cs. The FITC-Ac-SDKP was mainly localized into the HMVEC-Cs within 4 h in both non-irradiated controls (Fig. 7 a-c) and radiation-exposed cells (Fig. 7 e-g). In contrast, the FITC labelled scrambled peptide showed faint, non-discrete staining at the same concentrations (Fig. 7 i-k). Additionally, we performed FACS analysis to quantify the percentage of FITC-Ac-SDKP positive endothelial cells in non-irradiated, irradiated and irradiated-scrambled peptide respectively (Fig. 7 d, h and l). Irradiated HMVEC-Cs showed dose-dependent uptake of FITC-Ac-SDKP (r^2^ = 0.97; *p* < 0.001, *N* = 3) at peptide concentrations ranging from 0.9 μM (3.3 ± 0.7% FITC positive cells) to 14 μM (29.3 ± 2.7% FITC positive cells) (Fig. 7 m & n).

### In vivo uptake of labeled Ac-SDKP by endothelial cells

**Fig. 8:**
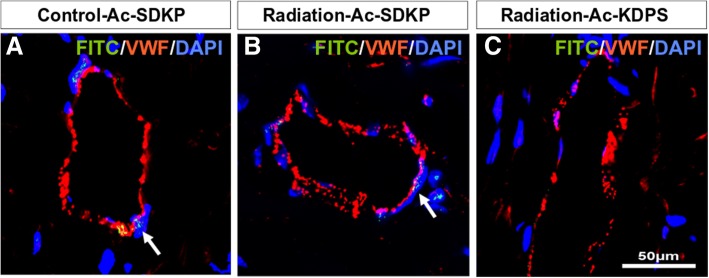
In vivo co-localization of FITC labeled Ac-SDKP with vWF staining: FITC-Ac-SDKP uptake in coronary endothelial cells were examined after 4 h of injections in mice with or without irradiation. Confocal images **a-c** show FITC-stained intracellular Ac-SDKP in green, Phycoerythrin (PE) labelled vWF as the endothelial marker in red and DAPI stained nuclei in blue. **a** shows co-localization of peptide with vWF in arteriolar wall in non-irradiated (control) mouse. Panel **b** represents tissue sections from radiation exposed mouse with arterial FITC-internalization in arteriolar endothelial cells. **c** shows scrambled peptide image. Scale bar: 50 μm, magnification × 400

We examined the uptake and distribution of FITC-Ac-SDKP in the mice heart following peptide injection in control and irradiated mice. Frozen tissue sections were stained with vWF antibody to identify the endothelial cells. In line with our in vitro peptide uptake assay results, green fluorescence staining was noted in the myocardium four hours after the injection of FITC-labeled Ac-SDKP in irradiated mice. A portion of green signal co-localized with the vWF staining, indicating endothelial cell uptake in controls and irradiated mice (Fig. 8 a-b). The labeled scrambled peptide (FITC-Ac-KDPS) did not demonstrate positive staining in this in vivo study (Fig. 8 c).

## Discussion

A major limiting factor of radiotherapy for cancers involving the chest and thoracic organs is the bystander damage to surrounding normal tissue, particularly the coronary vasculature. We have examined novel and previously unknown effects of thoracic ionizing radiation, and the protective effects of Ac-SDKP in resting myocardial blood flow. In addition, we addressed the potential causes of change in blood flow by examining vascular endothelium both in vitro and in vivo. Our study elucidates, for the first time, that the sustained deleterious effects of ionizing radiation on endothelial barriers involves cell loss and reduced endothelial expression of TJ and membrane scaffolding proteins including- claudin, and JAM-2. Ac-SDKP, a small peptide, freely localized to endothelial cells and inhibited radiation-induced ROS generation, and reconstituted the expression of TJP into the endothelium. Through these potential mechanisms, Ac-SDKP inhibited loss of endothelial cells from coronary arterial vasculature. In addition, we have demonstrated strong antifibrotic effects of Ac-SDKP to inhibit radiation-induced coronary vascular fibrosis.

### Endothelial cytotoxicity due to Cancer therapy

Most of the literature on cancer therapy induced endothelial cytotoxicity is derived from the chemotherapy protocols. For example, cardiac and endothelial toxicity of anthracyclines was attributed to redox activation of anthracyclines to semiquinone intermediates, which generate superoxide radicals upon reduction [30, 31]. One possibility for the loss of endothelial cell continuity could be apoptotic changes of the endothelial cells. However, we were not able to demonstrate apoptotic changes in our experiments. Lack of such changes could be due to the chronicity of our model (rats were sacrificed/studied at 18 weeks). Prior studies from Langley and associates showed radiation-induced apoptosis of microvascular endothelial cells as early as 6–10 h after irradiation [32]. In contrast to endothelial cells, our prior studies in cardiomyocytes demonstrated apoptotic changes 18-weeks after radiation exposure [22]. Unlike proliferative endothelial cells, cardiomyocytes are post-mitotic and often demonstrate gradual loss after radiation. Similarly, an increasing amount of clinical evidence shows that exposing the heart and epicardial coronary vessels to ionizing radiation significantly increases the risk of coronary artery diseases [33]. Long-term epidemiological studies conducted in Japanese atomic bomb survivors revealed that individuals who received an acute single dose of 1–2 Gy total-body irradiation showed remarkable increase in mortality from myocardial infarction [34]. More importantly, radiation-induced premature cardiovascular diseases are the commonest morbidities in survivors of Hodgkin’s disease [35] and breast cancer [36, 37], treated with high-dose radiation. A recent study by Hahn and associates examined the predictive role of dosimetric variables other than mean heart dose for the induction of late cardiotoxicity. Their coronary artery-based predictive model (e.g., volume of left anterior descending artery receiving 5 Gy and volume of left circumflex artery receiving 20 Gy) was superior to the whole heart model for the prediction of ischemic cardiac events [38].

Despite these alarming effects, no therapeutic agents are currently available to counteract these life-threatening effects. This novel studies shows a therapeutic potential of our lead molecule, Ac-SDKP in counteracting radiation-induced coronary vascular injury.

### Coronary vascular integrity and role of tight junction proteins

Endothelial cell integrity is crucial for physiological functions of coronary arteries by regulating ion diffusion, endothelium-dependent vasodilation and regulating chemotaxis, inflammation and fibrosis. Potential causes of radiation-induced endothelial damage include acute oxidative injury to the endothelial cells, followed by loss of endothelial cell barrier due to the direct effects of radiation on endothelial TJP and scaffolding proteins. Claudins, the major constituents of tight junction complexes, associates with occludins to establish the intercellular barriers to control the flow of ions, small proteins and vasoactive amines [39, 40]. These 20–27 kDa transmembrane proteins span the cell membrane 4 times and have PDZ-binding domains of membrane scaffold proteins [41]. In our study, abundant expression of caludin-1 and claudin-3 were noted in coronary vessels, and their expression was found to be reduced after radiation exposure. Ac-SDKP therapy, at least in part, reconstituted the downregulated TJPs, and inhibited endothelial cell loss from the coronary vessels. Although, it is still unclear whether the observed loss of endothelial cells is due to reduced cytopoiesis, apoptosis, or secondary to detachment from the basement membrane. TJ modulation in endothelial cells could significantly impact vascular permeability and leukocyte extravasation at inflammatory sites. Additionally, TJ modulation in endothelial cells may result in loss of cell polarity and abnormal shear stress, and these pathophysiological responses lead to vascular malformations and vessel fragility [42].

Tight junctions also serve the major functional purpose of providing a *barrier* and a *fence* within the membrane, by regulating cellular permeability. A previous study by Diserbo and associates showed that non-lethal total body irradiation produced an early transient increase in blood-brain-barrier (BBB) permeability in rats [43]. In 2017, Garg et al showed that radiation exposure causes segment-specific alterations in the expression of tight junction-related proteins, and speculated that interruption of the tight junction may be a key factor contributing to the endothelial permeability and cell barrier [44]. In our model, in addition to claudins, a significant reduction of ZO-1, one of the most studied junctional adaptor proteins, was noted after radiation exposure. ZO-1, also known as tight junction protein-1, is a 220 kD peripheral membrane scaffold protein which cross-links and anchors tight junction strand proteins to the actin cytoskeleton [45, 46].

In addition to the proteins involved in endothelial cell attachment and integrity, we have also noted the reduced expression of an immoglobulin superfamily protein, JAM-2. JAM-2 acts as an adhesive ligand for immune interactions and lymphocyte cell homing [47]. Ac-SDKP therapy reconstituted JAM-2 expression suggesting a crucial role of this peptide in preserving endothelial cell adhesion and anchorage, and also showed its potential role in lymphocyte homing and activation. The exact mechanisms explaining the radiation-induced regulation of tight junction assembly and scaffolding proteins are beginning to be elucidated, but our study underscores important therapeutic implications of Ac-SDKP for the endothelial cell attachment and vascular integrity.

### Radiation-induced ROS generation

ROS are a group of highly reactive chemicals under tight control of intracellular antioxidants [48]. ROS are not only mediators of oxidative stress, but also players of immune regulation [49]. Since endothelial cells are needed to orchestrate the early stages of inflammation and thrombosis, ROS-dependent endothelial dysfunction can generate pro-inflammatory and pro-thrombotic state in the coronary arteries. Because ROS-dependent endothelial injury plays crucial role in radiation-induced cardiovascular diseases, targeting them with a novel therapeutic agent is of great significance. In 2008, Lin et al reported increased oxidative stress, inflammation and expression of intercellular adhesion molecule mRNA expression in response to neurohumoral stimulation of large vessels [18]. Ac-SDKP prevented these effects without altering blood pressure or aortic hypertrophy. In our model, we have demonstrated that exposure to ionizing radiation is associated with excess H_2_O_2_ production by endothelial cells, which is inhibited by Ac-SDKP in a dose-dependent manner. Ac-SDKP-dependent endothelial ROS inhibition can be a potential mechanism to explain the anti-inflammatory and anti-fibrotic effects of this peptide in various oxidative stress models.

### Fibrotic remodeling of the coronary arteries

Prior studies have shown an association between oxidative stress and fibrosis [50]. Fibroblasts play a crucial role for the pathogenic remodeling of the blood vessels. These effects are exerted via direct effects on matrix regeneration and paracrine effects on vascular remodeling [51]. While replacement fibrosis is an expected phenomenon, novel therapeutic targets would be of tremendous benefit to prevent accelerated fibrotic remodeling induced by radiation. Prior in vitro studies and studies on hypertensive models have shown the antifbrotic effects of Ac-SDKP [16, 17, 52]. Most of the prior research on cardiac fibrosis is focused on neurohumoral stimulation. Since Ac-SDKP is a specific substrate for angiotension converting enzyme (ACE), one can reconcile the therapeutic benefits of the Ac-SDKP to be mediated by increased Ac-SDKP levels. Unlike ACE-inhibitors, Ac-SDKP has no reported hypotensive or nephrotoxic effects, while it still exerts strong anti-inflammatory and anti-fibrotic effects. Recovery from excess fibrotic response or reverse remodeling is an important therapeutic endpoint for the inhibition of radiation-induced cardiovascular morbidities. We have now demonstrated the antifibrotic effects of Ac-SDKP in relation to radiation-induced coronary vascular fibrosis. Overall, using an endogenous and non-toxic peptide like Ac-SDKP for the prevention of radiation-induced fibrotic remodeling of the coronary arteries is of strong translational significance to prevent radiation-induced heart disease.

### Limitations

We have utilized a rodent model of single high-dose (30 Gy) radiation exposure to the cardiac silhouette in this study. Stereotactic single-fraction radiotherapy had been used in the past in the select group of patients with stage I non-small lung cancers, that offered high local control with a reduced overall treatment time [53]. Single fraction to 30–34 Gy has been shown to equivalent to 3 and 4 fractions in randomized multi-institutional trials [54]. Also, single fraction has been shown to be equivalent to 5 fractions in a multi-institutional case-matched review [55]. Patients with more advanced stage III lung cancers are still treated with fractionated radiation therapy (60 Gy in 30 fractions) and in this group heart dose is associated with overall survival [56]. However, current cancer therapy protocols and guidelines use multi-dose fractionated radiation protocol for commonest cancers. Therefore, future studies utilizing fractionated dose of radiation exposure are needed for better clinical translation of these data. Additionally, given the prolonged natural history and delayed presentation of the sequel of radiation-induced microvascular diseases, it would be preferable to follow up the rodents for longer than 18-weeks.

## Conclusions and therapeutic implications

These studies confirm the previously reported detrimental effects of ionizing radiation on endothelial cell attachment and intercellular tight junction assembly. Furthermore, we provided evidence that Ac-SDKP, an endogenous peptide derived from thymosin-β4, is protective against these effects. Dysregulation of cell adhesion and tight junction assembly along with reactive oxidative injury are likely responsible for the endothelial cell loss and coronary macro- and microvasculature dysfunction. Further mechanistic studies of cell attachment and intercellular barrier function are currently in progress. Considering the serious cardiovascular side effects of radiation exposure, understanding the molecular mechanism of endothelial and vascular integrity would be important for minimizing risk. It is also important to recognize the fate of endothelial cell clearance mechanisms after radiation exposure. Most importantly, it is warranted to further examine the potential benefits of our lead molecule Ac-SDKP so as to determine whether this merits progression to future translational studies.

## Additional files

Additional file 1: **Figure S1.** Terminal deoxynucleotidyl transferase dUTP nick end labeling (TUNEL) staining for apoptosis detection. **Panel A** to **C** show the representative images of TYNEL staining of rat coronary vessels in myocardial sections. Apoptotic endothelial cells are not identified on Tunnel staining. **Panel A**: Non-radiated baseline control; **Panel B**: Radiation; and **Panel C**: Radiation + Ac-SDKP treated rat. **Panel D:** Positive control (arrow pointed to show apoptotic myocyte nucleus). *N* = 8–10, Scale bar: 20 μm, magnification × 400. (DOCX 609 kb)

Additional file 2: **Figure S2.** Effects of radiation and Ac-SDKP therapy on the mRNA expression of tight-junction molecules: Post-radiation rat heart tissue were used for mRNA analysis of common tight junction genes including *claudin-3* and *5, occludin* and *ZO-1*. Radiation exposure significantly reduced the gene expression of *claudin-3* and *ZO-1* in rat cardiac tissues (*, *p* < 0.001 for *claudin-3 and p = 0.01 for ZO-1* compared as radiation vs. controls), which was partially restored by Ac-SDKP treatment (†, *p* = 0.02 for *claudin-3* and *p* = 0.07 for *ZO-1* compared as radiation vs. radiation + Ac-SDKP). N = 8–10 each group. Rad, radiation. (DOCX 45 kb)
