# Supplementary material for: Effects of a novel peptide Ac-SDKP in radiation-induced coronary endothelial damage and resting myocardial blood flow
Source: Cardiooncology. 2018 Dec 18;4:8. doi: 10.1186/s40959-018-0034-1 (PMC6497419; doi:10.1186/s40959-018-0034-1)
Supplement: Supplementary file 1 — Figure S1. Terminal deoxynucleotidyl transferase dUTP nick end labeling (TUNEL) staining for apoptosis detection. Panel A to C show the representative images of TYNEL staining of rat coronary vessels in myocardial sections. Apoptotic endothelial cells are not identified on Tunnel staining. Panel A: Non-radiated baseline control; Panel B: Radiation; and Panel C: Radiation + Ac-SDKP treated rat. Panel D: Positive control (arrow pointed to show apoptotic myocyte nucleus). N = 8–10, Scale bar: 20 μm, magnification × 400. (DOCX 609 kb) [file 40959_2018_34_MOESM1_ESM.docx]

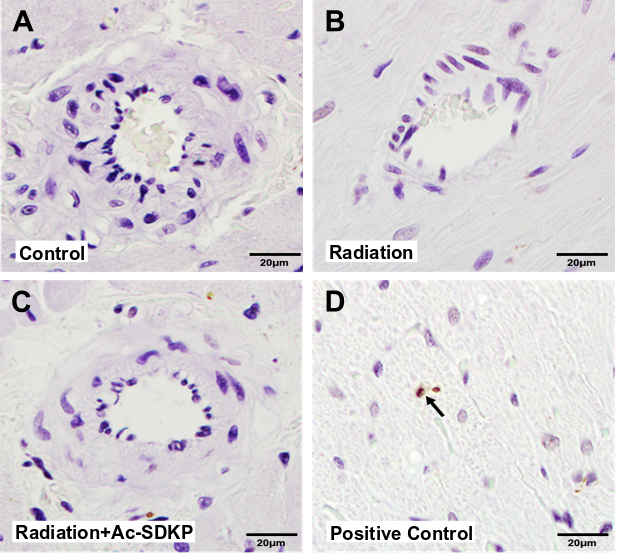


**Figure S1: Terminal deoxynucleotidyl transferase dUTP nick end labeling (TUNEL) staining for apoptosis detection. Panel A** to **C** show the representative images of TYNEL staining of rat coronary vessels in myocardial sections. Apoptotic endothelial cells are not identified on Tunnel staining. **Panel A**: Non-radiated baseline control; **Panel B**: Radiation; and **Panel C**: Radiation + Ac-SDKP treated rat. **Panel D:** Positive control (arrow pointed to show apoptotic myocyte nucleus). N=8-10, Scale bar: 20µm, magnification x 400.
