# Supplementary material for: Effects of a novel peptide Ac-SDKP in radiation-induced coronary endothelial damage and resting myocardial blood flow
Source: Cardiooncology. 2018 Dec 18;4:8. doi: 10.1186/s40959-018-0034-1 (PMC6497419; doi:10.1186/s40959-018-0034-1)
Supplement: Supplementary file 2 — Figure S2. Effects of radiation and Ac-SDKP therapy on the mRNA expression of tight-junction molecules: Post-radiation rat heart tissue were used for mRNA analysis of common tight junction genes including claudin-3 and 5, occludin and ZO-1. Radiation exposure significantly reduced the gene expression of claudin-3 and ZO-1 in rat cardiac tissues (*, p < 0.001 for claudin-3 and p = 0.01 for ZO-1 compared as radiation vs. controls), which was partially restored by Ac-SDKP treatment (†, p = 0.02 for claudin-3 and p = 0.07 for ZO-1 compared as radiation vs. radiation + Ac-SDKP). N = 8–10 each group. Rad, radiation. (DOCX 45 kb) [file 40959_2018_34_MOESM2_ESM.docx]

**
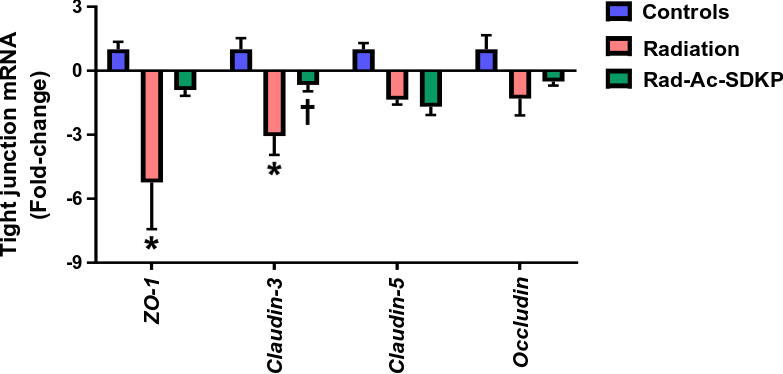
**

**Figure S2: Effects of radiation and Ac-SDKP therapy on the mRNA expression of tight-junction molecules:** Post-radiation rat heart tissue were used for mRNA analysis of common tight junction genes including *claudin-3* and *5, occludin* and *ZO-1*. Radiation exposure significantly reduced the gene expression of *claudin-3* and *ZO-1* in rat cardiac tissues (*, p <0.001 for *claudin-3 and p =0.01 for ZO-1* compared as radiation *vs*. controls), which was partially restored by Ac-SDKP treatment (†, p =0.02 for *claudin-3* and p= 0.07 for *ZO-1* compared as radiation *vs*. radiation + Ac-SDKP). N = 8-10 each group. Rad, radiation.
